# Presence of bone marrow micro‐metastases in stage I‐III colon cancer patients is associated with worse disease‐free and overall survival

**DOI:** 10.1002/cam4.1056

**Published:** 2017-04-12

**Authors:** Carsten T. Viehl, Benjamin Weixler, Ulrich Guller, Salome Dell‐Kuster, Rachel Rosenthal, Michaela Ramser, Vanessa Banz, Igor Langer, Luigi Terracciano, Guido Sauter, Daniel Oertli, Markus Zuber

**Affiliations:** ^1^ Department of SurgeryHospital Center BielBiel/BienneSwitzerland; ^2^ Department of SurgeryUniversity Hospital BaselBaselSwitzerland; ^3^ Department of SurgeryCantonal Hospital OltenOltenSwitzerland; ^4^ Department of Oncology/HematologyCantonal Hospital St. GallenSt. GallenSwitzerland; ^5^ University Clinic for Visceral Surgery and MedicineInselspital BerneUniversity of BerneBerneSwitzerland; ^6^ Basel Institute for Clinical Epidemiology and Biostatistics cebUniversity Hospital BaselBaselSwitzerland; ^7^ Department of SurgeryLindenhof HospitalBerneSwitzerland; ^8^ Department of PathologyUniversity Hospital BaselBaselSwitzerland; ^9^ Department of PathologyUniversity Hospital Hamburg‐EppendorfHamburgGermany

**Keywords:** Bone marrow, colon cancer, micro‐metastases, prognosis

## Abstract

The prognostic significance of bone marrow micro‐metastases (BMM) in colon cancer patients remains unclear. We conducted a prospective cohort study with long‐term follow‐up to evaluate the relevance of BMM as a prognostic factor for disease free (DFS) and overall survival (OS) in stage I‐III colon cancer patients. In this prospective multicenter cohort study 144 stage I‐III colon cancer patients underwent bone marrow aspiration from both iliac crests prior to open oncologic resection. The bone marrow aspirates were stained with the pancytokeratin antibody A45‐B/B3 and analyzed for the presence of epithelial tumor cells. DFS and OS were analyzed using a Cox proportional hazard model and robust standard errors to account for clustering in the multicenter setting. Median overall follow‐up was 6.2 years with no losses to follow‐up, and 7.3 years in patients who survived. BMM were found in 55 (38%) patients. In total, 30 (21%) patients had disease recurrence and 56 (39%) patients died. After adjusting for known prognostic factors, BMM positive patients had a significantly worse DFS (hazard ratio [HR] 1.33; 95% confidence interval [95% CI]: 1.02‐1.73; *P* = 0.037) and OS (HR 1.30; 95% CI: 1.09‐1.55; *P* = 0.003) compared to BMM negative patients. Bone marrow micro‐metastases occur in over one third of stage I‐III colon cancer patients and are a significant, independent negative prognostic factor for DFS and OS. Future trials should evaluate whether node‐negative colon cancer patients with BMM benefit from adjuvant chemotherapy.

## Background

The prognosis of colorectal cancer patients has markedly improved over the last three decades because of enhanced surgical resection techniques, new multimodal therapy strategies and advancements in chemotherapy. In particular, 5‐year survival rate of rectal cancer patients has improved from 48% to 68% whereas survival of colon cancer patients did not increase accordingly (from 51% to 65%) 1. This implies that rectal cancer patients nowadays show better disease outcomes than colon cancer patients 1. Indeed up to 40% of stage I‐III colon cancer patients develop disease recurrence within 5 years after operation 2. As these patients showed no signs of distant metastases at the time of operation, disease recurrence is most likely a result of occult tumor cell dissemination prior to surgery. This concept has recently been demonstrated in breast cancer patients, where dissemination of tumor cells into the blood stream as well as into the bone marrow (BM) has been identified as a source of disease recurrence 3, 4. Especially in early stage breast cancer patients these results are, however, still conflicting 5, 6. Indeed most data on circulating tumor cells and bone marrow micro‐metastases (BMM) are currently available for breast cancer patients. However, some evidence suggests that circulating tumor cells in the blood may have a prognostic significance in colorectal cancer patients as well 7, 8, 9. The role of BMM in colorectal cancer patients, however, is less clear, as there is only limited evidence from investigations with mostly short follow‐up periods indicating a poorer prognosis for bone marrow positive patients 10, 11, 12, 13, 14, 15, 16. Furthermore, the interpretation and comparison of these data is virtually impossible, as the studies were based on different protocols, used different antibodies and often analyzed colon and rectal cancer patients together. However, since there are significant differences in treatment strategies as well as in survival improvement over the last decades between colon and rectal cancer patients, colon and rectal cancer should be considered as distinct disease entities, necessitating separate analysis.

Therefore, the objective of the present study was to evaluate the long‐term prognostic significance of BMM in stage I‐III colon cancer patients.

## Patients and Methods

### Study design

This prospective study was conducted at three academic and university‐affiliated institutions in Switzerland (University Hospital Basel, Hospital Center Biel/Bienne, Cantonal Hospital Olten) between May 2000 and December 2006. The study protocol was in accordance with the respective institutional guidelines for experimental investigation with human subjects as well as with the Declaration of Helsinki and was approved by the respective ethical committees of all participating centers. The study was registered at ClinicalTrials.gov (NCT00826579). The in‐ and exclusion criteria of the study have been previously reported 17. Briefly, all patients with biopsy proven stage I‐III colon cancer were eligible for study inclusion. Exclusion criteria were rectal cancers, stage IV disease, prior abdominal cancer surgery, history of other solid malignancies, documented allergy to isosulfan blue as well as pregnancy and breast feeding. Written informed consent was obtained from all patients before surgery.

Standard oncologic en‐bloc resection (with appropriate lymphadenectomy) of the respective colon segment was performed. The primary tumor was staged according to the 6th version of the tumor‐node‐metastasis (TNM) classification system 18. Adjuvant chemotherapy was offered to stage III patients and to stage II patients featuring high risk factors (i.e. less than 12 lymph nodes analyzed, lymphovascular or perineural invasion, T4 tumor, poorly differentiated histology, or tumor perforation) as decided upon at the multidisciplinary tumor board conference. The participants of the conference were blinded for the BM results, and BMM were therefore not taken into account for the decision whether to offer adjuvant chemotherapy or not. The follow‐up period was calculated from the date of surgery. A structured surveillance was performed according to national guidelines for surveillance after curative colon cancer resection 19, 20. Follow‐up examinations were carried out according to national guidelines for surveillance after curative colon cancer resection in all patients at 3 months intervals during the first year and at 6 months intervals thereafter, for a period of 5 years. The Swiss national guidelines for surveillance after curative colon cancer resection demand CT‐scans (thorax/abdomen) in all patients at defined intervals of 12 months for a total of 5 years and CEA titers every three months for the first year and every six months for the second and third year. Subsequently, regular colonoscopy at 5 yearly intervals were performed. Development of local recurrence, distant metastases and death were continuously recorded. Contact was maintained with each patient's general practitioner until the end of the study period to ensure complete follow‐up.

### Bone marrow collection and definitions

After induction of general anesthesia and prior to surgery, BM was aspirated from both iliac crests (5 mL each). The BM specimens were then processed as described previously by our group 5. If one or more tumor cells were detected, the BM was considered positive. The pathologist was blinded regarding the primary tumor histology.

### Statistical analyses

Baseline characteristics of all patients included in the analysis are summarized descriptively using median and interquartile range (IQR) or number and percentages. A separate baseline table is presented in the appendix for the patients excluded from the study (Table S1, online only).

To estimate the effect of the BMM on disease free and overall survival, we plotted Kaplan Meier curves and used a Cox proportional hazard model taking into account the most relevant confounders. We used robust standard errors to adjust for potential clustering in the three study centers. The observation period in the overall survival (OS) analysis started on the day of surgery and lasted until the date of death irrespective of cause (failure) or until the last follow‐up visit (censoring). The observation period in the disease‐free survival (DFS) analysis started on the day of surgery and lasted until death irrespective of cause or the date of diagnosis of a recurrence, whichever occurred first, or until the last follow‐up visit (censoring). In the disease‐free survival analysis, recurrence or death due to any cause were considered as failure. The same observation period was considered in the analysis for the time to recurrence, in which recurrences or cancer‐related deaths were considered as the event of interest and non‐cancer‐related deaths were considered as a competing event. We estimated subdistributional hazard ratios in a semi‐parametric model according to Fine and Gray and plotted the cumulative incidence function. Moreover, cause‐specific hazard ratios were estimated for the event of interest (recurrence or cancer‐related death) as well as for the competing event (not‐cancer‐related death) in a Cox proportional hazard model, each with censoring the other event (i.e. not‐cancer‐related death was censored in the cause‐specific model for the event of interest and recurrence or cancer‐related death were censored in the cause‐specific model for the competing event, respectively). For these models a limited number of confounders (as compared to the model for OS and DFS) were taken into account due to the low number of events. Model diagnostics were performed investigating the proportional (subdistributional) hazard assumption and plotting scaled Schoenfeld residuals over time. All analyses were conducted using Intercooled Stata Version 13.1 for Mac (StataCorp, College Station, TX).

## Results

### Patients characteristics

**Figure 1:**
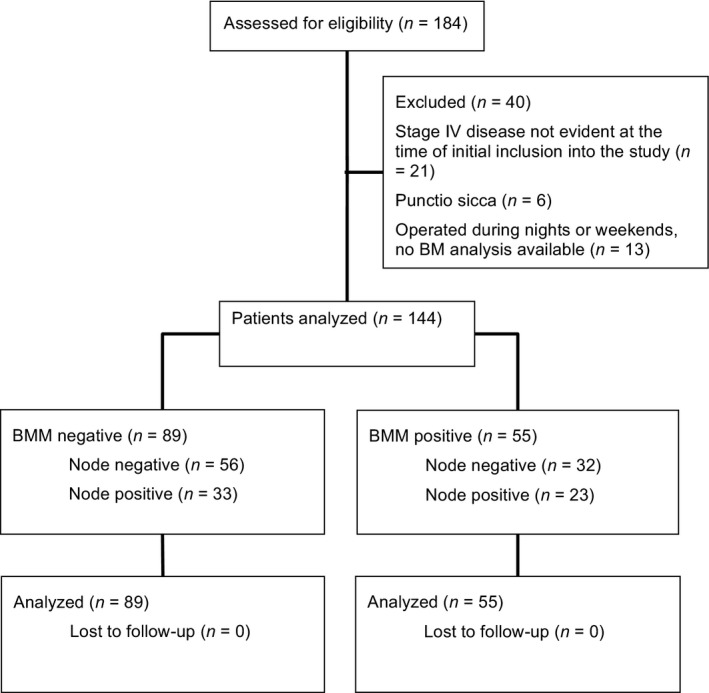
CONSORT diagram. BMM, bone marrow micro‐metastases.

Overall, 184 patients agreed to participate in the study. A total of 144 patients finally met the inclusion criteria. The study overview is shown in Figure 1. Clinical data and histological findings of the included patients are listed in Table 1.

**Table 1:** Baseline characteristics of all patients included in the analysis (*n* = 144)

	Total *n*=144	BM negative *n*= 89 (62%)	BM positive *n*= 55 (38%)
Age in years, median (IQR)	73.5 (66–79)	73 (66–78)	74 (66–81)
Gender, *n* (%)			
Male	76 (53%)	47 (53%)	29 (53%)
Female	68 (47%)	42 (47%)	26 (47%)
BMI in kg/m^2^, median (IQR)*	26.0 (23.0– 28.4)	25.7 (22.8–28.4)	26.6 (23.7–28.6)
Localisation, *n* (%)			
Right colon	61 (42%)	33 (37%)	28 (51%)
Left colon	24 (17%)	17 (19%)	7 (13%)
Sigmoid colon	59 (41%)	39 (44%)	20 (36%)
Tumor stage, *n* (%)			
pT1	11 ( 8%)	7 (8%)	4 (7%)
pT2	22 (15%)	13 (15%)	9 (16%)
pT3	94 (65%)	59 (66%)	35 (64%)
pT4	17 (12%)	10 (11%)	7 (13%)
Nodal status, *n* (%)			
pN0	88 (61%)	56 (63%)	32 (58%)
pN1	37 (26%)	21 (24%)	16 (29%)
pN2	19 (13%)	12 (13%)	7 (13%)
Lymphovascular invasion°, *n* (%)			
Absent	114 (80%)	73 (82%)	41 (76%)
Present	29 (20%)	16 (18%)	13 (24%)
Tumor grading, *n* (%)			
G1	‐‐	‐‐	‐‐
G2	107 (74%)	71 (80%)	36 (65%)
G3	37 (26%)	18 (20%)	19 (35%)
Centre, *n* (%)			
1	89 (62%)	55 (62%)	34 (62%)
2	40 (28%)	26 (29%)	14 (25%)
3	15 (10%)	8 (9%)	7 (13%)

BMM = bone marrow micro‐metastases

BMI = body mass index

*3 missing values in BMI; °1 missing value in lymphovascular invasion

BMM were detected in 55 (38%) of the 144 patients. Of these BMM positive patients, 32 (58%) were node negative and 23 (42%) were node positive. The BM positivity rate showed little variation across the different AJCC stages (37% for stage I, 36% for stage II, 41% for stage III). A mean of 0.06 epithelial cells per 1*10^6^ bone marrow cells (range 0.010‐1.714/1*10^6^) were detected in the BM aspirates. In absolute numbers, between 1 and 95 epithelial cells were counted per patient in their respective BM aspirates (mean 2.5).

In average, 23 lymph nodes were harvested and analyzed per patient (range 7‐62). Only five patients showed less than 12 lymph nodes. In total, 48 (33%) patients received adjuvant chemotherapy (3.7% of patients in AJCC stage I, 18% in stage II, and 64% in stage III, respectively). In 36% of stage III patients, chemotherapy was not administered because of advanced patient age or patient refusal. Adjuvant chemotherapy in stage I and II colon cancer was recommended according to interdisciplinary discussions and individual evaluation with the respective patients, based on the best available evidence 21. Similarly, 21 (38%) of 55 BMM positive patients received adjuvant chemotherapy, with 10% of BMM positive patients in AJCC stage I, 18% in stage II, and 70% in stage III, respectively.

### Time to recurrence

**Figure 2:**
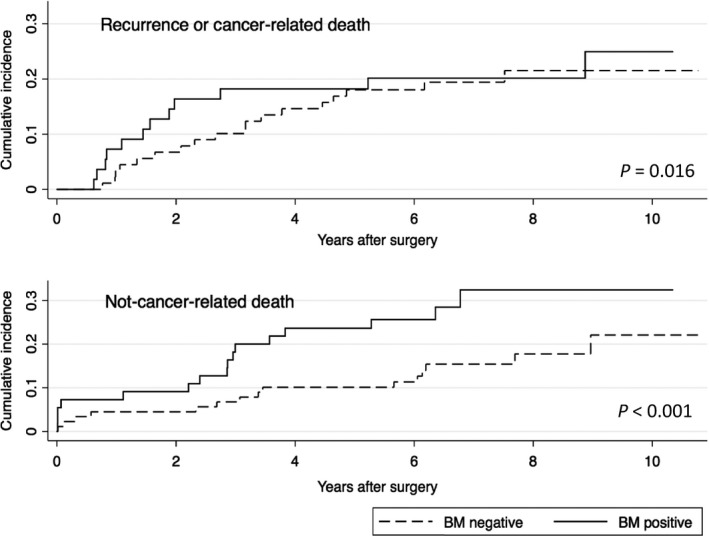
Cumulative incidence of recurrence or cancer‐related death (top) and not‐cancer related death for the covariates bone marrow (BM) negative and positive.

Follow‐up could successfully be completed in all 144 patients. During the follow‐up period a total of 30 patients had a tumor recurrence and/or died from recurrent disease. These events occurred in 12 (22%) of 55 BM positive patients and in 18 (20%) of 89 BM negative patients. Median follow‐up time for all patients was 6.2 years (IQR 3.0‐7.9 years). Median follow‐up time for patients without event of interest was 7.3 years (IQR 6.2‐10.8 years), and the corresponding follow‐up time for patients with a competing event was 2.9 years (IQR 0.6‐5.7 years). In Fine and Gray's multivariable subdistributional hazard model a positive BMM status was associated with a significant shorter time to recurrence (subdistributional hazard ratio [SHR] 1.17, 95% confidence interval [95%CI]: 1.03‐1.34, *P* = 0.016) (Fig. 2, Table 2). Further independent prognostic factors were male gender (SHR 2.77, 95% CI: 1.94‐7.92, *P* < 0.001) and positive nodal status (SHR 1.86,95% CI: 1.48‐2.35, *P* < 0.001). The cause‐specific hazard model for the event of interest shows consistent results (Table 2).

**Table 2:** Hazard models for time to recurrence, cancer‐related death and not‐cancer‐related death

	Univariate analysis		Multivariable analysis	
Exposures	SHR (95% CI)	*P*‐value	SHR (95% CI)	*P*‐value
Fine and Gray's subdistributional hazard model for time to recurrence				
BM pos. versus neg.	1.14 (0.82, 1.58)	0.439	1.17 (1.03, 1.34)	0.016
Age ‐ per 10 years increase	1.04 (0.61, 1.76)	0.896	1.03 (0.57, 1.86)	0.917
Male versus female	2.63 (1.97, 3.52)	<0.001	2.77 (1.94, 3.96)	<0.001
Tumor stage T3/4 versus T1/2	2.93 (0.92, 9.36)	0.070	2.73 (0.94, 7.92)	0.064
Nodal status N1/2 versus N0	2.29 (2.09, 2.50)	<0.001	1.86 (1.48, 2.35)	<0.001

	Univariate analysis		Multivariable analysis	
Exposures	HR (95% CI)	*P*‐value	HR (95% CI)	*P*‐value
Cause‐specific hazard model for recurrence and cancer‐related death				
BM pos. versus neg.	1.27 (0.97, 1.67)	0.087	1.23 (1.11, 1.36)	<0.001
Age ‐ per 10 years increase	1.12 (0.66, 1.91)	0.662	1.14 (0.61, 2.13)	0.691
Male versus female	2.58 (1.87, 3.56)	<0.001	2.50 (1.64, 3.80)	<0.001
Tumor stage T3/4 versus T1/2	2.85 (0.88, 9.27)	0.081	2.53 (0.88, 7.28)	0.085
Nodal status N1/2 versus N0	2.41 (2.09, 2.79)	<0.001	2.02 (1.64, 2.49)	<0.001
Cause‐specific hazard model for not‐cancer‐related death				
BM pos. versus neg.	2.12 (1.82, 2.47)	<0.001	1.72 (1.37, 2.15)	<0.001
Age ‐ per 10 years increase	2.78 (1.29, 5.96)	0.009	3.00 (1.18, 7.60)	0.021
Male versus female	0.92 (0.64, 1.34)	0.679	0.70 (0.62, 0.79)	<0.001
Tumor stage T3/4 versus T1/2	0.60 (0.40, 0.91)	0.017	0.36 (0.15, 0.86)	0.022
Nodal status N1/2 versus N0	1.04 (0.76, 1.42)	0.818	1.92 (1.16, 3.16)	0.011

SHR, subdistributional hazard ratio, HR, hazard ratio.

### Disease free survival analysis

**Figure 3:**
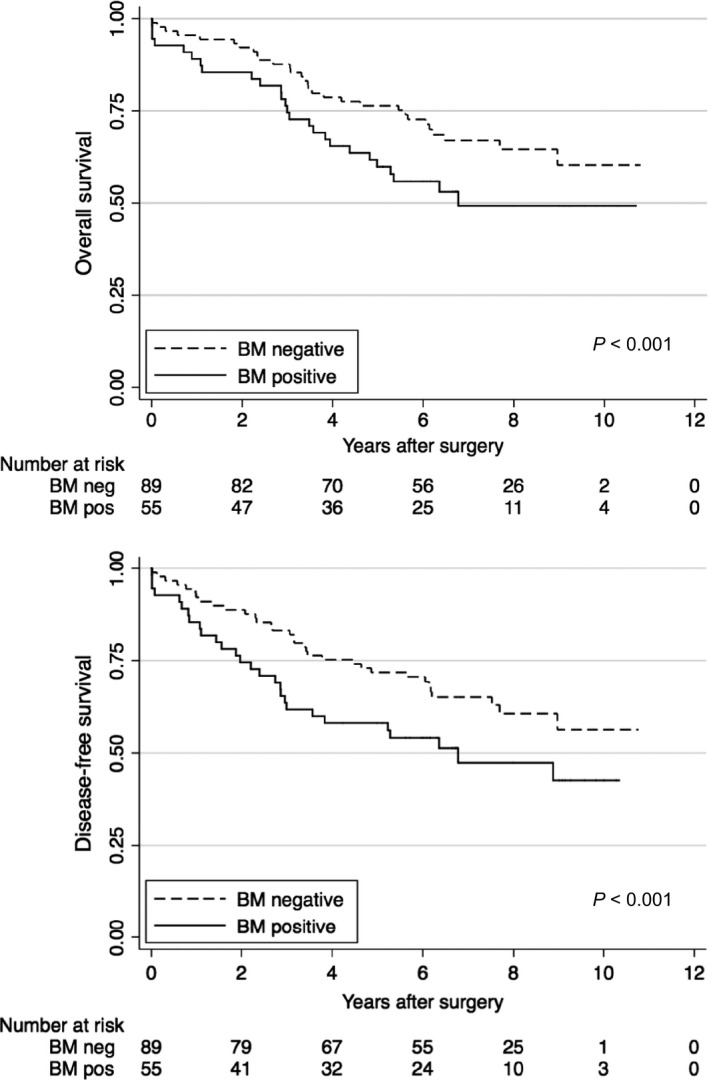
Kaplan–Meier survival curves for overall survival and disease‐free survival for the covariates bone marrow (BM) negative and positive.

For DFS analysis 61 (42%) events were recorded in 144 patients. These events occurred in 28 (51%) of 55 BMM positive patients and in 33 (37%) of 89 BMM negative patients. Median follow‐up time for DFS was 6.2 years (IQR 3.0‐7.9 years). Median follow‐up time for patients who survived (without recurrence) was 7.3 years (IQR 6.2‐9.1 years). Patients with BMM had a significant shorter DFS than patients without BMM in univariate analysis (*P* < 0.001, Fig. 3, Table 3).

**Table 3:** Uni‐ and multivariable Cox regression analysis for disease‐free survival (*n* = 143 due to one missing value for lymphovascular invasion, LVI)

	Univariate analysis		Multivariable analysis	
Exposures	HR (95% CI)	*P*‐value	HR (95% CI)	*P*‐value
BM pos. versus neg.	1.65 (1.54, 1.76)	<0.001	1.33 (1.02, 1.73)	0.037
Age ‐ per 10 years increase	1.67 (1.58, 1.76)	<0.001	1.76 (1.57, 1.96)	<0.001
Male versus female	1.49 (1.08, 2.04)	0.014	1.21 (0.94, 1.57)	0.146
Tumor stage T3/4 versus T1/2	1.09 (0.54, 2.20)	0.813	0.63 (0.30, 1.32)	0.219
Nodal status N1/2 versus N0	1.58 (1.41, 1.77)	<0.001	1.56 (1.11, 2.20)	0.011
Grade 3 versus 2	2.04 (1.19, 3.48)	<0.001	1.51 (0.74, 3.09)	0.259
LVI yes versus no	3.16 (2.21, 4.53)	<0.001	2.77 (1.87, 4.11)	<0.001

After adjusting for potential confounders in multivariable Cox regression analyses BMM was shown to be an independent negative prognostic factor for disease recurrence or death of any cause (HR 1.33, 95% CI: 1.02‐1.73, *P* = 0.037). Further independent negative prognostic factors for DFS were increased age (HR 1.76, 95% CI: 1.57‐1.96, *P* < 0.001), positive nodal status (HR 1.56, 95% CI: 1.11‐2.20, *P* = 0.011), and lymphovascular invasion (HR 2.77, 95% CI: 1.87‐4.11, *P* < 0.001) (Table 3).

### Overall survival analysis

A total of 56 (39%) deaths of any cause out of the 144 patients were recorded. Reasons for noncancer related deaths were cardial (*n* = 15), pulmonal (*n* = 6), other malignancy (*n* = 3), suicide (*n* = 1), cerebrovascular (*n* = 1), renal failure (*n* = 1), upper gastrointestinal bleeding (*n* = 2) and unknown (*n* = 4). Median follow‐up for OS was 6.2 years (IQR 3.8‐8.2 years). Median follow‐up time in those who survived was 7.3 years (IQR 6.2‐9.1 years). Patients with BMM had a significant shorter OS than patients without BMM (*P* < 0.001, Fig. 3, Table 4).

**Table 4:** Uni‐ and multivariable Cox regression analysis for overall survival (n = 143 due to one missing value for lymphovascular invasion, LVI)

	Univariate analysis		Multivariable analysis	
Exposures	HR (95% CI)	*P*‐value	HR (95% CI)	*P*‐value
BM pos. versus neg.	1.66 (1.35, 2.04)	<0.001	1.30 (1.09, 1.55)	0.003
Age ‐ per 10 years increase	1.88 (1.70, 2.11)	<0.001	2.04 (1.69, 2.46)	<0.001
Male versus female	1.38 (0.89, 2.09)	0.149	1.10 (0.79, 1.54)	0.573
Tumor stage T3/4 vs T1/2	1.02 (0.42, 2.50)	0.957	0.55 (0.21, 1.42)	0.218
Nodal status N1/2 versus N0	1.60 (1.31, 1.95)	<0.001	1.24 (1.68, 2.01)	<0.001
Grade 3 versus 2	2.00 (1.11, 3.60)	0.022	1.51 (0.71, 3.19	0.284
LVI yes versus no	3.06 (1.97, 4.74)	<0.001	2.81 (1.68, 4.71)	<0.001

After adjusting for potential confounders in multivariable Cox regression analyses, BMM remained a significant negative prognostic factor for OS (HR 1.30, 95% CI: 1.09‐1.55, *P* = 0.003). Further independent prognostic factors for OS were increased age (HR 2.04, 95% CI: 1.69‐2.46, *P* < 0.001), positive nodal stage (HR 1.24, 95% CI: 1.68‐2.01, *P* < 0.001) and lymphovascular invasion (HR 2.81, 95% CI: 1.68‐4.71, *P* < 0.001) (Table 4).

The differential effect of BMM in patients with pN0 and pN1/2 was estimated in a post hoc analysis (Table S2). The risk for disease‐free survival was higher in node positive patients (HR 1.71, 95% CI: 1.56‐1.87, *P* < 0.001) than in node‐negative patients (HR 1.27, 95% CI: 1.14‐1.41 *P* < 0.001). For overall survival, the added interaction term did not reach statistical significance.

## Discussion

The objective of the present prospective multicenter study was to evaluate the long‐term prognosis of BMM in colon cancer patients undergoing curative surgery. This investigation provides compelling evidence that BMM are an independent negative prognostic factor associated with a significant shorter time to recurrence, and with a significant shorter overall and disease free survival. This finding is of cardinal importance as BMM were detected in more than one third of stage I‐III colon cancer patients.

In the present investigation, BMM positive patients had a roughly 1.3‐fold increased risk of disease recurrence and death compared to patients with a negative BM. BMM positivity clearly had a detrimental effect on survival time. The presence of BMM even in lymph node negative colon cancer patients indicates metastatic spread early in the course of the disease. The earlier appearance of events in BMM positive patients could therefore refer to an advanced, but histopathologically yet undetected tumor stage at the time of operation.

Using immunocytochemistry with a pancytokeratin antibody, BMM were detected in 38% of our patients. These results are in accordance with BMM detection rates of other solid cancer types, which were reported to be around 35% 5, 14. However, BMM detection rates in studies with colorectal cancer patients show a wide range. This is mainly due to different antibodies or detection methods used. These relevant technical differences make in‐between comparisons of the reported results nearly impossible. While immunocytochemistry with the antibody A45‐B/B3 was performed in the present study which has already been successfully used by our group 5, others capitalized on magnetic activated cell sorting 22 or reverse transcription‐polymerase chain reaction (rt‐PCR), mainly of cytokeratin (CK)‐20 RNA 23, 24, 25. These different methods have already been compared: In their study on metastatic colorectal cancer patients, Vogelaar et al. used the A45‐B/B3 antibody as well as CK‐20 rt‐PCR and reported a relevant difference in detection rates between the two techniques (33% by A45‐B/B3 and 20% by CK‐20) 23. The CK‐20 detection rate was similarly low in the work by Koch et al. focusing on colorectal cancer patients with liver metastases. The authors therefore advocated the use of A45‐B/B3 24. In other studies, stage IV patients were included 12, 23, 25. The usefulness of BMM detection in already metastasized patients is, however, questionable as BMM are generally regarded as a sign of tumor cell dissemination that takes place prior to surgery and serves rather as an indicator of occult disease than of an obvious metastatic situation.

The prognostic value of BMM in colon and rectal cancer patients has been discussed controversially for years. The discussion first came up in the early 1990s with the introduction of immunocytochemical investigations with monoclonal antibodies that made the detection of histogenetically different cells possible. Beginning in the 1990s, first studies on colorectal cancer patients reported higher disease recurrence rates when BMM were present at time of surgery 26, 27. However, because of rather small sample sizes and short follow‐up times these studies did not have an impact on daily clinical practice. More recently, one group demonstrated a detrimental effect of BMM in a cohort of 235 colorectal cancer patients (with 32% rectal cancer patients) 26. The authors of this study, however, reported a very low BMM detection rate of 12% and 17%, using two different antibodies and detection techniques. In contrast to such suggestive evidence, O'Connor et al. could not detect an impact on DFS in their cohort of 34 colorectal cancer patients (seven with BMM) which is quite likely due to the small sample size 27. In their most recently published study, Vogelaar et al. could not detect a statistically significant association between disseminated tumor cells in the bone marrow and disease outcome in 125 patients with primary colorectal cancer. However, the BMM detection rate (18%) was significantly lower than in the here presented study and the smaller patient cohort may be responsible for the nonsignificant results 28. This is also reflected by a much wider 95% confidence interval for overall survival (0.45‐2.09) and disease‐free survival (0.35‐1.82) compared to our results. It is important to note that most studies did not distinguish between colon and rectal cancer patients. Only one study so far was focusing on colon cancer patients exclusively and the authors identified BMM as an independent negative prognostic factor for survival 29. However, this study included 20% stage IV patients receiving palliative surgery and the mean observation time of 39 months was rather short, preventing a clear statement on disease recurrence and OS. In this context the publication by Kienle et al. is relevant as the authors reported a significantly lower detection rate of BMM in rectal cancer patients after administration of neoadjuvant chemoradiation (16.7%) compared to patients without preoperative treatment (33%) 30. This finding strongly advocates for a clear separation of colon and rectal cancer patients in clinical trials, as many rectal cancer patients nowadays receive preoperative therapy and are biologically different with better prognosis compared to colon cancer. Interestingly, in the here presented study BMM were more common in patients with right‐sided colon cancer. There is an ongoing discussion if tumor localization itself has a prognostic meaning in patients with colon cancer. The majority of existing studies report poorer survival for right‐sided colon cancer, compared to left‐sided 31, 32. It has been speculated that a later detection due to the right‐sided localization or differences in embryologic origin and fecal exposure may play a role. A most recently published study however questions the right‐sided survival disadvantage as the authors identified that prognostic differences between right‐ and left‐sided colon cancer may not be real and seem to occur due to differences regarding confounders 33. It therefore remains a matter of debate if localization of the primary tumor will be of prognostic significance. However, due to the limited sample size the present study cannot answer why BMM appeared more often in patients with right sided colon cancer. This question should be explored in future research planned to investigate this hypothesis.

An important strength of our study is that we included only stage I–III colon cancer patients resulting in a much more homogeneous patient sample. To differentiate whether there is an association between nodal status and BMM, we performed a not‐prespecified explanatory analysis that provided some evidence that the negative effect of BMM on disease‐free survival seems to be more pronounced in patients with pN1/pN2 compared to pN0. However, tests for interaction have a low power and the interaction term did not reach statistical significance for overall survival (Table S2). This question has therefore to be further explored in a subsequent study.

Another major strength of the present study is its long follow‐up time exceeding 6 years without any patients who were lost to follow‐up.

However, we would also like to acknowledge the limitations of our study. A major drawback in clinical cancer studies lies in the possibility that competing events such as not‐cancer related death may produce misleading results. It is well known that competing events occur frequently in clinical trials and that the standard Cox proportional hazards model is not an entirely adequate analysis in the presence of such competing events. We therefore used a Fine and Gray subdistributional hazard model to estimate separately the risk for the event of interest and the competing event. Furthermore, the present investigation is a prospective cohort study and potential unmeasured confounding cannot be excluded. However, the baseline characteristics between included and excluded patients are comparable in our study and the presence of a relevant bias is therefore unlikely. Moreover, the sample size limited the statistical model in this study. To avoid collinearity, the effect of chemotherapy was therefore not included in the analysis.

And finally, genetic testing was not a state of the art procedure at the time of patient inclusion and we can therefore not report on these differences. This would have been interesting especially for the comparison of right‐ versus left‐sided colon cancer as discussed above.

In conclusion, our study provides compelling evidence that BMM are associated with a significant shorter time to recurrence, and with a significantly worse DFS and OS in stage I‐III colon cancer patients. Bone marrow micro‐metastases were identified as an independent negative prognostic factor for DFS and OS. This is particularly relevant as the presence of BMM represents a frequent phenomenon occurring in over one third of stage I‐III colon cancer patients. As aspiration of BM is simple, BM analysis for micro‐metastasis should be incorporated in future trials to evaluate whether node‐negative colon cancer patients with BMM benefit from adjuvant chemotherapy.

## Conflict of Interest

Rachel Rosenthal is an employee of F. Hoffmann‐La Roche Ltd. since May 01, 2014. The present study was conducted before Rachel Rosenthal joined F. Hoffmann‐La Roche Ltd. and has no connection to her employment by the company. Rachel Rosenthal continues to be affiliated with the University of Basel.

## Supporting information


**Table S1:** Baseline characteristics of all patients excluded from the analysis (*n* = 40): 21 patients had stage IV disease and in additional 19 patients no BM was harvested or usable (six punctio sicca, 11 patients operated during night or weekend, one patient refused bone marrow aspiration, one technical problem with analysis of bone marrow aspiration)

**Table S2**: Post hoc analysis of the multivariable model by adding the interaction term node negative vs. node positive^1^.
