# Supplementary material for: Presence of bone marrow micro‐metastases in stage I‐III colon cancer patients is associated with worse disease‐free and overall survival
Source: Cancer Med. 2017 Apr 12;6(5):918–27. doi: 10.1002/cam4.1056 (PMC5430093; doi:10.1002/cam4.1056)
Supplement: Supplementary file 1 — Table S1: Baseline characteristics of all patients excluded from the analysis (n = 40): 21 patients had stage IV disease and in additional 19 patients no BM was harvested or usable (six punctio sicca, 11 patients operated during night or weekend, one patient refused bone marrow aspiration, one technical problem with analysis of bone marrow aspiration) [file CAM4-6-918-s001.docx]

**Appendix Table A1:** Baseline characteristics of all patients excluded from the analysis (n=40): 21 patients had stage IV disease and in additional 19 patients no BM was harvested or usable (6 punctio sicca, 11 patients operated during night or weekend, one patient refused bone marrow aspiration, one technical problem with analysis of bone marrow aspiration)

|  | Total (n = 40) |
| --- | --- |
| Age in years, median (IQR) | 71 (65 - 76) |
| Gender, n (%) |  |
| Male | 24 (60%) |
| Female | 16 (40%) |
| BMI in kg/m^2^*, median (IQR) | 24.9 (22.8 - 28.1) |
| Localization, n (%) |  |
| Right colon | 20 (50%) |
| Left colon | 5 (13%) |
| Sigmoid colon | 15 (38%) |
| Tumor stage, n (%) |  |
| pT1 | 2 ( 5%) |
| pT2 | 4 (10%) |
| pT3 | 22 (55%) |
| pT4 | 12 (30%) |
| Nodal status, n (%) |  |
| pN0 | 2 (5%) |
| pN1 | 4 (10%) |
| pN2 | 22 (55%) |
| pN3 | 12 (30%) |
| Lymphovascular invasion°, n (%) |  |
| Absent | 22 (56%) |
| Present | 17 (44%) |
| Tumor grading, n (%) |  |
| G1 | 1 ( 3%) |
| G2 | 23 (58%) |
| G3 | 16 (40%) |
| Center, n (%) |  |
| 1 | 17 (43%) |
| 2 | 18 (45%) |
| 3 | 5 (13%) |

BMI = body mass index

* 2 missing values in BMI; ° 1 missing value in lymphovascular invasion
