# Supplementary material for: Presence of bone marrow micro‐metastases in stage I‐III colon cancer patients is associated with worse disease‐free and overall survival
Source: Cancer Med. 2017 Apr 12;6(5):918–27. doi: 10.1002/cam4.1056 (PMC5430093; doi:10.1002/cam4.1056)
Supplement: Supplementary file 2 — Table S2: Post hoc analysis of the multivariable model by adding the interaction term node negative vs. node positive1. [file CAM4-6-918-s002.docx]

Appendix 2: Post-hoc analysis of the multivariable model by adding the interaction term node negative vs. node positive^1^.

|  | Disease-free survival^1^ | | Overall survival^2^ | |
| --- | --- | --- | --- | --- |
| **Exposures** | **HR (95% CI)** | **p-value** | **HR (95% CI)** | **p-value** |
| BM pos. vs. neg. in patients with pN0 | 1.27 (1.14, 1.41) | <0.001 | 1.32 (1.22, 1.42) | p<0.001 |
| BM pos. vs. neg. in patients with pN1/2 | 1.71 (1.56, 1.87) | <0.001 | 1.62 (1.12, 2.35) | p=0.010 |

^1^Not-pre-specified analysis for DFS: Interaction term was added post-hoc to the multivariable analysis and did reach statistical significance for disease-free survival, 1.34 (1.15, 1.57; p<0.001)

^2^Not-pre-specified analysis for OS: Interaction term was added post-hoc to the multivariable analysis and did not reach statistical significance for overall survival, HR of 1.23 (0.81, 1.88; p=0.329).
